# Clinical course and characteristics of patients with coronavirus disease 2019 in Wuhan, China: a single-centered, retrospective, observational study

**DOI:** 10.18632/aging.103745

**Published:** 2020-08-24

**Authors:** Yanfang Liu, Lina Liu, Ye Wang, Xinyang Du, Hong Ma, Jing Yao

**Affiliations:** 1 Cancer Center, Union Hospital, Tongji Medical College, Huazhong University of Science and Technology, Wuhan 430022, People's Republic of China

**Keywords:** COVID-19, SARS-CoV-2, chronic diseases, risk factors, infection

## Abstract

Background: Severe acute respiratory syndrome coronavirus 2(SARS-CoV-2) is the virus responsible for the coronavirus disease 2019(COVID-19) pandemic. Despite the extensive studies aiming to understand the pathology of COVID-19, the clinicopathological characteristics and risk factors associated with COVID-19 remain mostly unclear. In this study, we assessed the clinical course and features of COVID-19 patients.

Findings: There were 59 patients (54.1%) that had no fever. One-hundred(91.7%) patients required oxygen therapy, which improved percutaneous oxygen saturation (SpO_2_). Seventy-two (66.1%) patients aged over 60; these patients were more likely to develop respiratory symptoms. Only 13(11.9%) patients were positive for anti-SARS-CoV-2 antibodies, SARS-CoV-2 nucleic acid, and computed tomography (CT) findings. We found significant differences in age, respiratory symptoms, and heart rates between patients with and without underlying conditions.

Conclusions: Our findings suggest that oxygen plays an important role in the treatment of COVID-19 patients and that age and underlying diseases are significant risk factors for COVID-19. Most COVID-19 patients have no fever, and CT provides higher detection rates than antibody- and nucleic acid-based detection methods.

Methods: We analyzed data from 109 confirmed COVID-19 cases. We compared the clinicopathological characteristic of patients stratified according to age and underlying diseases, as well as assessed the detection rates of different diagnostic methods.

## INTRODUCTION

In December 2019, an outbreak of severe acute respiratory syndrome coronavirus 2(SARS-CoV-2) in Wuhan, China, marked the beginning of the coronavirus disease 2019(COVID-19) pandemic [1–3]. As of April 8, 2020, the number of confirmed cases has risen to 1.35 million worldwide.[4] COVID-19 often present with persistent fever, cough, chest distress, dyspnea, and sore throat. Some patients also develop gastrointestinal symptoms, including diarrhea, while other patients have no obvious symptoms, making the virus spread containment extremely challenging [5, 6]. In COVID-19 patients, lung computed tomography (CT) findings include bilateral scattered patchy ground-glass density shadows and consolidation stripe shadows in both lungs [7, 8].

Despite the extensive efforts of the last months to understand the pathology of COVID-19 and identify therapeutic targets, the clinicopathological characteristics and risk factors associated with COVID-19 remain largely unclear. In this study, we investigated the clinicopathological characteristics and treatment outcomes in 109 patients diagnosed with COVID-19. The findings reported herein provide a better understanding of the clinical course, treatment efficacy, and risk factors in COVID-19 patients, providing a step forward toward the development of novel strategies to contain the pandemic.

## RESULTS

### Patient demographics and characteristics

In this study, we included 109 patients diagnosed with COVID-19 from February 13, 2020, to February 29, 2020, at Wuhan Union Hospital. The demographics and characteristics of these patients are summarized in Supplementary Table 1. The median age of all patients was 63 (range, 29-97), and 72 (66.1%) patients were aged over 60. There were 51 (46.8%) female patients and 58 (53.2%) male patients. Fourth-seven (43.1%) patients had chronic diseases. Among all patients, 100 (91.7%) required oxygen therapy, after which percutaneous oxygen saturation (SpO_2_) values returned to physiological levels (SpO_2_ ≥ 94%).

### Clinical features

The clinical features of COVID-19 patients are summarized in Supplementary Table 2. Common symptoms included increased heart rate (n = 57; 52.3%), cough (n = 56; 51.4%), mild fever (37.3°C-38°C), and chest tightness (n = 36; 33.0%); high fever (39°C-40°C) was observed in three patients (2.8%). Only 50 (45.9%) patients presented with fever, while the remaining 59 (54.1%) patients did not develop fever throughout the disease course. Among the patients who developed fever, the median body temperature was 37.8°C (ranges, 37.3°C -40°C), and the median fever duration was 2.5 days (ranges, 1-8 days). 95 (87.1%) patients presented with respiratory symptoms at the time of diagnosis, and in 31 (28.5%) patients, respiratory symptoms continued even after O_2_ supplement. Of the 47 (43.1%) patients who had chronic diseases, 41 (87.2%) had respiratory symptoms at the time of diagnosis, and 13 (31.7%) had respiratory symptoms after oxygen therapy. A total of 31 (28.4%) patients were diagnosed with abnormal SpO_2_; in these patients, SpO_2_ values returned to physiological levels after O_2_ supplement.

Among all patients with underlying diseases, 17 (36.2%) were O_2_ unsaturated on admission. The median age of patients with respiratory symptoms after oxygen therapy was 65.5 (ranges, 29-97), whereas the median age of patients without respiratory symptoms after O_2_ supplement was 62 (range, 29-91). The median age of patients with respiratory symptoms requiring oxygen therapy was 73 (range, 60-83), whereas that of patients not requiring oxygen therapy was 65 (range, 58-65) (Supplementary Table 1).

In this study, we also compared the demographics and clinical characteristics of patients with and without chronic diseases (Table 1). While the median age of patients with chronic diseases was 69 (range, 38-97), that of patients without underlying conditions was 60 (range, 29-91) (Table 1); this difference was statistically significant. Compared with patients with chronic diseases, respiratory symptoms were less frequent, and heart rates were lower in patients without underlying conditions (*P*<0.0284 and *P*<0.0001, respectively; Table 1). No significant differences in gender or other clinical characteristics were observed between patients with chronic diseases and those without chronic conditions (Table 1). Significant factors identified by univariate analyses (age, respiratory symptoms, and heart rate) were included in multivariate analyses; age and heart rate were identified as significant risk factors of chronic diseases (Table 1).

**Table 1:** Unvariate and multivariate analysis of patients with or without chronic diseases.

	**All patients**	**Chronic diseases**	**Non-chronic diseases**	**Univariate P value**	**Multivariate P value**
**Sex**	109(100)	47(43.1)	62(56.9)		
Female	51(46.8)	20(18.3)	31(28.4)	0.4403	
Male	58(53.2)	27(24.6)	31(28.4)		
**Age, median(range)**	63(29-97)	69(38-97)	60(29-91)	<0.0001	<0.0001
≤39	6(5.5)	1(0.9)	5(4.6)	0.0061	
40-59	31(28.4)	7(6.4)	24(22.0)		
60-79	56(51.4)	28(25.7)	28(25.7)		
≥80	16(14.6)	11(10.0)	5(4.6)		
**Initial respiratory symptoms^a^**					
Yes	95(87.2)	41(37.6)	54(49.5)	0.9831	
No	14(12.8)	6(5.5)	8(7.3)		
**Respiratory symptoms after O_2_ supplement**					
Yes	31(28.4)	18(16.5)	23(21.1)	0.2676	
No	69(63.3)	23(21.1)	46(42.2)		
**Median temperature, °C**	37.2(36.6-40)	37.2(36.6-39.8)	37.2(36.8-40)	0.6853	
**Initial** **median SpO2 value (%)**	95(64-98)	95(91-97)	102(86-135)	0.4961	
**Median SpO2 value after O_2_ supplement (%)**	98(96-100)	98(96-100)	98(96-100)	0.9876	
**Median heart rate, beats per min**	101(84-135)	100(84-126)	95(80-98)	<0.0001	<0.0001
**Median respiratory rate, breaths per min**	22(20-34)	22(20-34)	21(20-28)	0.0284	

Data are n (%), unless otherwise specified. Abbreviations: COVID-19, coronavirus disease 2019; SpO2, Percutaneous oxygen saturation; O_2_, oxygen.

^a^including cough, sore throat, short of breath, chest tightness, expectoration and dyspnea.

### Laboratory parameters and imaging findings

**Figure 1:**
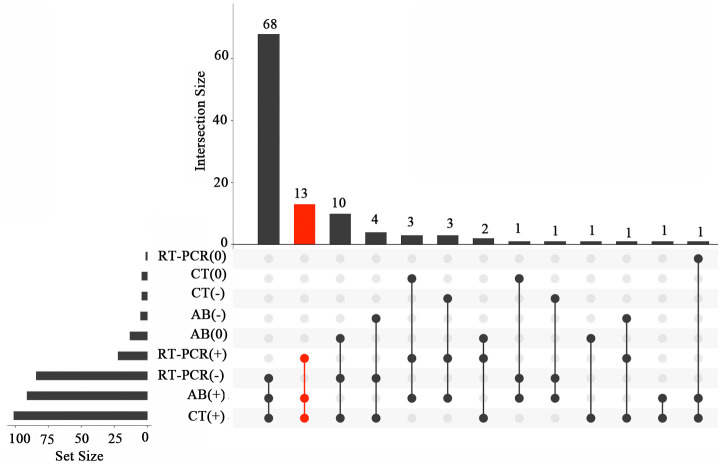
**Venn diagram showing the laboratory parameters and CT findings in COVID-19 patients.** AB (+), positive antibody assay; AB (-), negative antibody assay; AB (0), antibody assay was not performed; RT-PCR (+), positive RT-PCR assay; RT-PCR (-), negative RT-PCR assay; RT-PCR (0), RT-PCR assay was not performed; CT (+), positive CT diagnosis; CT (-), negative CT diagnosis; CT (0), CT was not performed.

The results of laboratory examination and computed tomography (CT) in COVID-19 patients are shown in Figure 1 and Supplementary Table 3. Although 101 (92.6%) of the patients had positive CT findings, and 91 (83.5%) were positive for anti-SARS-CoV-2 antibodies, only 24 (22.0%) were positive for SARS-CoV-2 nucleic acid. Only 13 (11.9%) of the cases were positive for anti-SARS-CoV-2 antibodies, SARS-CoV-2 nucleic acid, and CT findings. Eighty-three (76.1%) cases were positive for both anti-SARS-CoV-2 antibodies and CT findings, and 18 (16.5%) were positive for SARS-CoV-2 nucleic acid and CT findings.

Eighty-two (75.3%) of the patients with anti-SARS-CoV-2 antibodies presented with respiratory symptoms at the time of diagnosis, whereas 9 (8.2%) had no respiratory symptoms at diagnosis (Table 2). Eighty-eight (80.7%) patients with CT findings had respiratory symptoms at the time of diagnosis, while 13 (11.9%) cases with CT findings had no respiratory symptoms at diagnosis. Importantly, the number of patients who had respiratory symptoms after oxygen therapy was lower among individuals with anti-SARS-CoV-2 antibodies and CT findings. Among the patients with anti-SARS-CoV-2 antibodies and who received O_2_ supplement, 24 (29.3%) had respiratory symptoms even after oxygen therapy. Additionally, among the patients with CT findings and who received O_2_ supplement, 27 (29.0%) had respiratory symptoms after oxygen therapy. However, among the patients with positive SARS-CoV-2 RT-PCR results, respiratory symptoms continued after oxygen therapy in 12 (52.2%) of them.

**Table 2:** Examinations and clinical symptoms of patients with COVID-19.

	**All patients**	**Initial respiratory symptoms^a^**		**Initial SpO2 value (%)**		**Supplemental O_2_**		**Respiratory symptoms after O_2_ supplement**		**SpO2 value after O_2_ supplement (%)**		**Respiratory symptoms without O_2_ supplement**		**SpO2 value without O_2_ supplement (%)**	
		**Yes**	**No**	**≥94**	**<94**	**Yes**	**No**	**Yes**	**No**	**≥94**	**<94**	**Yes**	**No**	**≥94**	**<94**
**Antibody assay^b^**															
Positive	91(83.5)	82(75.3)	9(8.2)	68(62.4)	23(21.1)	82(75.3)	9(8.2)	24(22.0)	58(53.2)	82(75.3)	0(0)	1(0.9)	8(7.3)	9(8.2)	0(0)
Negative	5(4.6)	3(2.8)	2(1.8)	3(2.8)	2(1.8)	5(4.6)	0(0)	1(0.9)	4(3.7)	5(4.6)	0(0)	0(0)	0(0)	0(0)	0(0)
Not performed	13(11.9)	10(9.1)	3(2.8)	7(6.4)	6(5.5)	13(11.9)	0(0)	6(5.5)	7(6.4)	13(11.9)	0(0)	0(0)	0(0)	0(0)	0(0)
**RT-PCR assay^c^**															
Positive	24(22.0)	22(20.2)	2(1.8)	17(15.5)	7(6.4)	23(21.1)	1(0.9)	12(11.0)	11(10.1)	23(21.1)	0(0)	0(0)	1(0.9)	1(0.9)	0(0)
Negative	84(77.1)	72(66.1)	12(11.0)	60(55.0)	24(22.1)	76(69.7)	8(7.3)	19(17.4)	57(52.3)	76(69.7)	0(0)	1(0.9)	7(6.4)	8(7.3)	0(0)
Not performed	1(0.9)	1(0.9)	0(0)	1(0.9)	0(0)	1(0.9)	0(0)	0(0)	1(0.9)	1(0.9)	0(0)	0(0)	0(0)	0(0)	0(0)
**CT diagnosis**															
Positive	101(92.6)	88(80.7)	13(11.9)	72(66.1)	29(26.6)	93(85.3)	8(7.3)	27(24.8)	66(60.6)	93(85.3)	0(0)	1(0.9)	7(6.4)	8(7.3)	0(0)
Negative	4(3.7)	3(2.8)	1(0.9)	3(2.8)	1(0.9)	3(2.8)	1(0.9)	2(1.8)	1(0.9)	3(2.8)	0(0)	0(0)	1(0.9)	1(0.9)	0(0)
Not performed	4(3.7)	4(3.7)	0(0)	3(2.8)	1(0.9)	4(3.7)	0(0)	2(1.8)	2(1.8)	4(3.7)	0(0)	0(0)	0(0)	0(0)	0(0)

Data are n (%), unless otherwise specified. Abbreviations: COVID-19, coronavirus disease 2019; SpO2, Percutaneous oxygen saturation; O_2,_ oxygen.

^**a**^ including cough, sore throat, short of breath, chest tightness, expectoration and dyspnea; ^b^Anti-SARS-CoV-2 antibody assay; ^c^SARS-CoV-2 RT-PCR assay.

## DISCUSSION

SARS-CoV-2 is the third coronavirus discovered so far; it is considerably more infectious than SARS-CoV and MERS-CoV [9–12], leading to the rapid spread of COVID-19 across almost every country [13, 14]. In this study, we reported on the clinicopathological characteristics and clinical course of 109 COVID-19 patients. The median age was 63 (ranges 29-97), and 72 (66.1%) patients aged over 60. Forty-seven (43.1%) patients had underlying conditions. Among all patients, 100 (91.7%) received oxygen therapy, after which SpO_2_ values returned to physiological levels, suggesting that O_2_ supplementation played an important role in improving the condition of patients infected with SARS-CoV-2.

Interestingly, more than half of the patients (54.1%) did not develop fever, in contrast to the study by Goyal P et al., which reported that 77.1% of patients had a fever [15]. Interestingly, Zhiliang Hu et al. reported that only 20.8% of COVID-19 patients developed fever [16], highlighting the high variation in the symptoms of COVID-19 across cohorts. We believe that the fact that SARS-CoV-2 has been circulating for more than half a year has contributed to the attenuation of the symptoms caused by the virus. Additionally, as some COVID-19 patients remain asymptomatic, routine testing using antibody detection tests, nucleic acid testing, and chest CT, should be implemented in every country to contain the pandemic. Among patients who had a fever, the median body temperature was 37.8°C, and the median duration of the fever was 2.5 days; therefore, SARS-CoV-2 infection screening solely by measuring body temperature is insufficient. Additionally, a few patients did not have respiratory symptoms on admission but developed such symptoms later during the disease course. This finding highlights the need for early thorough clinical examination, CT scan, and laboratory testing in suspected cases. Moreover, self-isolation for at least 14 days is crucial for the prevention of community spread of SARS-CoV-2.

In our cohort, more than half of COVID-19 patients were elderly (over 60 years old), consistent with findings from previous studies [1, 17]; hence, we conclude that elderly patients are more likely to be infected with SARS-CoV-2 and that strict measures should be implemented to prevent the spear of the virus in the elderly. Our findings also revealed that people over 60 years old were more likely to develop respiratory symptoms, including abnormal SpO_2_, compared with younger individuals. Furthermore, the elderly were less likely to improve after oxygen therapy, further supporting the need for close monitoring of COVID-19 patients aged more than 60. We observed significant differences in age, respiratory symptoms, and heart rates between patients with chronic diseases and those without underlying conditions, suggesting that these factors may indicate the presence of chronic diseases.

It has been reported that patients with underlying diseases were more likely to contract SARS-CoV-2 [17, 18]. In this study, we found that 43.1% of the patients had chronic diseases. After oxygen therapy, the respiratory symptoms continued in one-third of the patients with underlying conditions. However, SpO_2_ returned to the physiological levels in all patients after oxygen therapy, pinpointing the importance of O_2_ supplement for the treatment of COVID-19 patients with underlying conditions.

The combination of laboratory examination and CT scans plays an important role in the diagnosis of COVID-19. In this cohort, although 92.6% of patients received CT-based diagnosis, and 83.5% were positive for anti-SARS-CoV-2 antibodies, only 22.0% of them were positive for SARS-CoV-2 nucleic acid. Importantly, only 11.9% of the patients were positive for anti-SARS-CoV-2 antibodies, SARS-CoV-2 nucleic acid, and CT diagnosis. Previous studies have shown that some COVID-19 patients were negative for anti-SARS-CoV-2 antibodies and viral nucleic acid at early stages [19]. CT provided a higher detection rate than laboratory examination, highlighting the importance of CT imaging to confirm SARS-CoV-2 infection.

There are several limitations to this study. First, the patient cohort consisted of only 109 cases, but the patients’ characteristics were similar to those in previous studies [1, 6, 18]. Second, we did not test for hematological indicators of heart, liver, and kidney function. Additionally, we did not assess for important observation indexes, such as clinical outcomes, medication plans, living conditions, and less common symptoms, which might be vital for clinical decision making and outcome prediction.

In conclusion, our findings suggest that oxygen therapy plays an important role in the treatment of COVID-19 patients. Old age and underlying diseases are the main risk factors of SARS-CoV-2 infection; therefore, the elderly and individuals with chronic diseases should be closely monitored after contracting the virus. Most COVID-19 patients have no fever; hence, thorough clinical examination, CT, and laboratory examination are pivotal for COVID-19 diagnosis. Additionally, the detection rate of CT is superior to anti-SARS-CoV-2 antibody testing and SARS-CoV-2 RT-PCR; thus, CT imaging should be implemented in the clinical practice to confirm SARS-CoV-2 infection.

## MATERIALS AND METHODS

### Study design and participants

In this study, we analyzed data from 109 COVID-19 patients admitted to the Wuhan Union Hospital in Hubei, China, from February 13, 2020, to February 29, 2020, according to the WHO Interim Guidelines [20]. The study was approved by the Ethics Committee of Wuhan Union Hospital. Informed consent was provided by all patients.

### Data collection

We collected the following data from 109 patients diagnosed with SARS-CoV-2 infection: age, gender, respiratory symptoms (fever, cough, dyspnea, chest tightness, sore throat), vital signs at admission (temperature, heart rate, respiratory rate, SpO_2_), chronic medical history (chronic heart disease, coronary heart disease, hypertension, hyperlipidemia, diabetes), treatment (O_2_ therapy), respiratory symptoms after treatment (fever, cough, dyspnea, chest tightness, sore throat), SpO_2_ after treatment, presence of anti-SARS-CoV-2 antibodies, SARS-CoV-2 RT-PCR assay results, and CT findings. Continuous variables were expressed as medians and ranges, whereas categorical variables were expressed as numbers and percentages (%).

### Statistical analysis

Differences among groups were analyzed using Fisher’s exact test, χ² test, univariate analysis, and multivariate analysis according to the type of data. Statistical analyses were conducted using GraphPad Prism 8.0 and TBtools software. *P*-values <0.05 were considered statistically significant.

## Supplementary Material

Supplementary Table 2

Supplementary Table 1

Supplementary Table 3
