# Supplementary material for: Clinical course and characteristics of patients with coronavirus disease 2019 in Wuhan, China: a single-centered, retrospective, observational study
Source: Aging (Albany NY). 2020 Aug 24;12(16):15946–53. doi: 10.18632/aging.103745 (PMC7485711; doi:10.18632/aging.103745)
Supplement: Supplementary Table 2 [file aging-12-103745-s002..doc]

**Supplementary Table 2. Clinical features of patients with COVID-19.**

|  | **All patients** | **Initial respiratory symptoms^a^** | | **Initial SpO2 value (%)** | | **Supplemental O_2_** | | **Respiratory symptoms after O_2_ supplement** | | **SpO2 value after O_2_ supplement (%)** | | **Respiratory symptoms without O_2_ supplement** | | **SpO2 value without O_2_ supplement (%)** | |
| --- | --- | --- | --- | --- | --- | --- | --- | --- | --- | --- | --- | --- | --- | --- | --- |
|  |  | **Yes** | **No** | **≥94** | **<94** | **Yes** | **No** | **Yes** | **No** | **≥94** | **<94** | **Yes** | **No** | **≥94** | **<94** |
| **Temperature,median (range),℃** | 37.2(36.6-40) | 37.2(36.6-40) | 37.15(36.8-38.5) | 37.1(36.6-40) | 37.5(36.8-39.8) | 37.2(36.8-40) | 37.1(36.6-37.8) | 37.2(36.8-38.7) | 37.2(36.8-40) | 37.2(36.8-40) | - | 36.9 | 37.15(36.6-37.8) | 37.1(36.6-37.8) | - |
| ≤37.2 | 59(54.1) | 49(44.9) | 10(9.2) | 51(46.8) | 8(7.3) | 52(47.7) | 7(6.4) | 16(14.7) | 36(33.0) | 52(47.7) | 0(0) | 1(0.9) | 6(5.5) | 7(6.4) | 0(0) |
| 37.3-38 | 36(33.0) | 33(30.3) | 3(2.7) | 19(17.4) | 17(15.6) | 34(31.2) | 2(1.8) | 12(11.0) | 22(20.2) | 34(31.2) | 0(0) | 0(0) | 2(1.8) | 2(1.8) | 0(0) |
| 38.1-39 | 11(10.1) | 10(9.2) | 1(0.9) | 7(6.4) | 4(3.7) | 11(10.1) | 0(0) | 2(1.8) | 9(8.3) | 11(10.1) | 0(0) | 0(0) | 0(0) | 0(0) | 0(0) |
| 39-40 | 3(2.8) | 3(2.8) | 0(0) | 1(0.9) | 2(1.8) | 3(2.8) | 0(0) | 0(0) | 3(2.8) | 3(2.8) | 0(0) | 0(0) | 0(0) | 0(0) | 0(0) |
| **Heart rate, /min** | 101(84-135) | 101(84-135) | 100.5(89-115) | 101(84-128) | 102(89-135) | 101(84-135) | 101(88-118) | 97(84-128) | 103(86-135) | 101(84-135) | - | 90 | 101.5(88-118) | 101(88-118) | - |
| 60-100 | 52(47.7) | 45(41.3) | 7(6.4) | 37(33.9) | 15(13.8) | 48(44.0) | 4(3.7) | 21(19.3) | 27(24.7) | 48(44.0) | 0(0) | 1(0.9) | 3(2.8) | 4(3.7) | 0(0) |
| >100 | 57(52.3) | 50(45.9) | 7(6.4) | 41(37.6) | 16(14.7) | 52(47.7) | 5(4.6) | 9(8.2) | 43(39.5) | 52(47.7) | 0(0) | 0(0) | 5(4.6) | 5(4.6) | 0(0) |
| **Initial SpO2 value (%)** | 95(64-98) | 95(64-98) | 95(92-98) | 95(94-98) | 92(64-93) | 95(64-98) | 96(94-98) | 94(76-97) | 95(64-98) | 95(64-98) | - | 96 | 96(94-98) | 96(94-98) | - |
| 94-100 | 78(71.6) | 66(60.6) | 12(11.0) | 78(71.6) | 0(0) | 69(63.4) | 9(8.2) | 19(17.4) | 50(45.9) | 69(63.4) | 0(0) | 1(0.9) | 8(7.3) | 9(8.2) | 0(0) |
| 80-93 | 27(24.7) | 25(22.9) | 2(1.8) | 0(0) | 27(24.7) | 27(24.7) | 0(0) | 10(9.2) | 17(15.5) | 27(24.7) | 0(0) | 0(0) | 0(0) | 0(0) | 0(0) |
| 70-79 | 3(2.8) | 3(2.8) | 0(0) | 0(0) | 3(2.8) | 3(2.8) | 0(0) | 2(1.8) | 1(0.9) | 3(2.8) | 0(0) | 0(0) | 0(0) | 0(0) | 0(0) |
| ≤69 | 1(0.9) | 1(0.9) | 0(0) | 0(0) | 1(0.9) | 1(0.9) | 0(0) | 0(0) | 1(0.9) | 1(0.9) | 0(0) | 0(0) | 0(0) | 0(0) | 0(0) |
| **Initial respiratory symptoms** |  |  |  |  |  |  |  |  |  |  |  |  |  |  |  |
| No | 14(12.8) |  | 14(12.8) | 12(11.0) | 2(1.8) | 11(10.1) | 3(2.8) | 2(1.8) | 9(8.2) | 11(10.1) | 0(0) | 0(0) | 3(2.8) | 3(2.8) | 0(0) |
| Cough | 56(51.4) | 56(51.4) |  | 42(38.5) | 14(12.8) | 51(46.8) | 5(4.6) | 17(15.5) | 39(35.8) | 51(46.8) | 0(0) | 1(0.9) | 4(3.7) | 5(4.6) | 0(0) |
| Sore throat | 9(8.2) | 9(8.2) |  | 9(8.2) | 0(0) | 7(6.4) | 2(1.8) | 3(2.8) | 4(3.7) | 7(6.4) | 0(0) | 0(0) | 2(1.8) | 2(1.8) | 0(0) |
| Short of breath | 28(25.7) | 28(25.7) |  | 16(14.7) | 12(11.0) | 28(25.7) | 0(0) | 7(6.4) | 21(19.3) | 28(25.7) | 0(0) | 0(0) | 0(0) | 0(0) | 0(0) |
| Chest tightness | 36(33.0) | 36(33.0) |  | 22(20.2) | 14(12.8) | 35(32.1) | 1(0.9) | 12(11.0) | 23(21.1) | 35(32.1) | 0(0) | 0(0) | 1(0.9) | 1(0.9) | 0(0) |
| Expectoration | 17(15.5) | 17(15.5) |  | 10(9.2) | 7(6.4) | 15(13.8) | 2(1.8) | 5(4.6) | 10(9.2) | 15(13.8) | 0(0) | 0(0) | 2(1.8) | 2(1.8) | 0(0) |
| Dyspnea | 6(5.5) | 6(5.5) |  | 5(4.6) | 1(0.9) | 5(4.6) | 1(0.9) | 0(0) | 5(4.6) | 5(4.6) | 0(0) | 1(0.9) | 0(0) | 1(0.9) | 0(0) |

Data are n (%), unless otherwise specified. Abbreviations: COVID-19, coronavirus disease 2019; SpO2, Percutaneous oxygen saturation; O_2,_ oxygen.

**^a^** including cough, sore throat, short of breath, chest tightness, expectoration and dyspnea.
