# Supplementary material for: Clinical course and characteristics of patients with coronavirus disease 2019 in Wuhan, China: a single-centered, retrospective, observational study
Source: Aging (Albany NY). 2020 Aug 24;12(16):15946–53. doi: 10.18632/aging.103745 (PMC7485711; doi:10.18632/aging.103745)
Supplement: Supplementary Table 1 [file aging-12-103745-s001..doc]

**Supplementary Table 1. Demographics characteristics of patients with COVID-19.**

|  | **All patients** | **Initial respiratory symptoms^a^** | | **Initial SpO2 value (%)** | | **Supplemental O_2_** | | **Respiratory symptoms after O_2_ supplement** | | **SpO2 value after O_2_ supplement (%)** | | **Respiratory symptoms without O_2_ supplement** | | **SpO2 value without O_2_ supplement (%)** | |
| --- | --- | --- | --- | --- | --- | --- | --- | --- | --- | --- | --- | --- | --- | --- | --- |
|  |  | **Yes** | **No** | **≥94** | **<94** | **Yes** | **No** | **Yes** | **No** | **≥94** | **<94** | **Yes** | **No** | **≥94** | **<94** |
| **Sex** | 109(100) | 95(87.1) | 14(12.9) | 78(71.6) | 31(28.4) | 100(91.7) | 9(8.3) | 31(28.5) | 69(63.2) | 100(91.7) | 0(0) | 1(0.9) | 8(7.4) | 9(8.3) | 0(0) |
| Female | 51(46.8) | 40(36.7) | 11(10.1) | 38(34.9) | 13(11.9) | 46(42.2) | 5(4.6) | 15(13.8) | 31(28.4) | 46(42.2) | 0(0) | 1(0.9) | 4(3.7) | 5(4.6) | 0(0) |
| Male | 58(53.2) | 55(50.4) | 3(2.8) | 40(36.7) | 18(16.5) | 54(49.5) | 4(3.7) | 16(14.7) | 38(34.8) | 54(49.5) | 0(0) | 0(0) | 4(3.7) | 4(3.7) | 0(0) |
| **Age, median(range)** | 63(29-97) | 63(29-97) | 64(55-87) | 62(29-97)) | 68(40-91) | 62.5(29-97) | 69(58-83) | 65.5(29-97) | 62(29-91) | 62.5(29-97) | - | 73(60-83) | 65(58-65) | 70.5(58-83) | - |
| ≤39 | 6(5.5) | 6(5.5) | 0(0) | 6(5.5) | 0(0) | 6(5.5) | 0(0) | 2(1.8) | 4(3.7) | 6(5.5) | 0(0) | 0(0) | 0(0) | 0(0) | 0(0) |
| 40-49 | 11(10.1) | 11(10.1) | 0(0) | 8(7.3) | 3(2.8) | 11(10.1) | 0(0) | 3(2.8) | 8(7.3) | 11(10.1) | 0(0) | 0(0) | 0(0) | 0(0) | 0(0) |
| 50-59 | 20(18.3) | 17(15.5) | 3(2.8) | 15(13.7) | 5(4.6) | 19(17.4) | 1(0.9) | 4(3.7) | 15(13.7) | 19(17.4) | 0(0) | 0(0) | 1(0.9) | 1(0.9) | 0(0) |
| 60-69 | 40(36.7) | 34(31.2) | 6(5.5) | 30(27.6) | 10(9.1) | 36(33.0) | 4(3.7) | 13(11.9) | 23(21.1) | 36(33.0) | 0(0) | 0(0) | 4(3.7) | 4(3.7) | 0(0) |
| 70-79 | 16(14.7) | 14(12.9) | 2(1.8) | 13(11.9) | 3(2.8) | 13(11.9) | 3(2.8) | 4(3.7) | 9(8.2) | 13(11.9) | 0(0) | 1(0.9) | 2(1.8) | 3(2.8) | 0(0) |
| ≥80 | 16(14.7) | 13(11.9) | 3(2.8) | 6(5.5) | 10(9.1) | 15(13.7) | 1(0.9) | 6(5.5) | 9(8.2) | 15(13.7) | 0(0) | 0(0) | 1(0.9) | 1(0.9) | 0(0) |
| **Chronic illness** | 47(43.1) | 41(37.6) | 6(5.5) | 30(27.6) | 17(15.5) | 41(37.6) | 6(5.5) | 13(11.9) | 28(25.7) | 41(37.6) | 0(0) | 1(0.9) | 5(4.6) | 6(5.5) | 0(0) |
| Hypertension | 38(34.9) | 33(30.3) | 5(4.6) | 22(20.2) | 16(14.7) | 33(30.3) | 5(4.6) | 18(16.5) | 15(13.8) | 33(30.3) | 0(0) | 1(0.9) | 4(3.7) | 5(4.6) | 0(0) |
| Diabetes | 25(22.9) | 23(21.1) | 2(1.8) | 15(13.7) | 10(9.2) | 25(22.9) | 0(0) | 11(10.1) | 14(12.8) | 25(22.9) | 0(0) | 0(0) | 0(0) | 0(0) | 0(0) |
| Hyperlipemia | 1(0.9) | 1(0.9) | 0(0) | 1(0.9) | 0(0) | 1(0.9) | 0(0) | 1(0.9) | 0(0) | 1(0.9) | 0(0) | 0(0) | 0(0) | 0(0) | 0(0) |
| Coronary heart disease | 1(0.9) | 1(0.9) | 0(0) | 1(0.9) | 0(0) | 1(0.9) | 0(0) | 1(0.9) | 0(0) | 1(0.9) | 0(0) | 0(0) | 0(0) | 0(0) | 0(0) |
| Cardiac disease | 1(0.9) | 1(0.9) | 0(0) | 1(0.9) | 0(0) | 0(0) | 1(0.9) | 0(0) | 0(0) | 0(0) | 0(0) | 0(0) | 1(0.9) | 1(0.9) | 0(0) |

Data are n (%), unless otherwise specified. Abbreviations: COVID-19, coronavirus disease 2019; SpO2, Percutaneous oxygen saturation; O_2_, oxygen.

**^a^** including cough, sore throat, short of breath, chest tightness, expectoration and dyspnea.
