# Supplementary material for: Clinical course and characteristics of patients with coronavirus disease 2019 in Wuhan, China: a single-centered, retrospective, observational study
Source: Aging (Albany NY). 2020 Aug 24;12(16):15946–53. doi: 10.18632/aging.103745 (PMC7485711; doi:10.18632/aging.103745)
Supplement: Supplementary Table 3 [file aging-12-103745-s003..pdf]

SUPPLEMENTARY TABLE

Supplementary Table 3. Laboratory examination and CT diagnosis of patients with COVID-19.

	All patients	Antibody assay positive	Antibody assay negative	Antibody assay not performed	RT-PCR assay positive	RT-PCR assay negative	RT-PCR assay not performed	CT diagnosis positive	CT diagnosis negative	CT diagnosis not performed
Antibody assay[a]										
Positive	91(83.5)	91(83.5)	0(0)	0(0)	20(18.3)	70(64.3)	1(0.9)	83(76.1)	4(3.7)	4(3.7)
Negative	5(4.6)	0(0)	5(4.6)	0(0)	1(0.9)	4(3.7)	0(0)	5(4.6)	0(0)	0(0)
Not performed	13(11.9)	0(0)	0(0)	13(11.9)	3(2.8)	10(9.1)	0(0)	13(11.9)	0(0)	0(0)
RT-PCR assay[b]										
Positive	24(22.0)	20(18.3)	1(0.9)	3(2.8)	24(22.0)	0(0)	0(0)	18(16.5)	3(2.8)	3(2.8)
Negative	84(77.1)	70(64.3)	4(3.7)	10(9.1)	0(0)	84(77.1)	0(0)	82(75.2)	1(0.9)	1(0.9)
Not performed	1(0.9)	1(0.9)	0(0)	0(0)	0(0)	0(0)	1(0.9)	1(0.9)	0(0)	0(0)
CT diagnosis										
Positive	101(92.6)	83(76.1)	5(4.6)	13(11.9)	18(16.5)	82(75.2)	1(0.9)	101(92.6)	0(0)	0(0)
Negative	4(3.7)	4(3.7)	0(0)	0(0)	3(2.8)	1(0.9)	0(0)	0(0)	4(3.7)	0(0)
Not performed	4(3.7)	4(3.7)	0(0)	0(0)	3(2.8)	1(0.9)	0(0)	0(0)	0(0)	4(3.7)

Data are n (%), unless otherwise specified. Abbreviations: COVID-19, coronavirus disease 2019; CT, computed tomography; SARS-CoV-2, severe acute respiratory syndrome coronavirus 2; RT-PCR, real-time quantitative polymerase chain reaction.
[a]Anti-SARS-CoV-2 antibody assay; [b]SARS-CoV-2 RT-PCR assay.
